# S100B polymorphisms are associated with age of onset of Parkinson’s disease

**DOI:** 10.1186/s12881-018-0547-3

**Published:** 2018-03-12

**Authors:** Camilla Fardell, Anna Zettergren, Caroline Ran, Andrea Carmine Belin, Agneta Ekman, Olof Sydow, Lars Bäckman, Björn Holmberg, Nil Dizdar, Peter Söderkvist, Hans Nissbrandt

**Affiliations:** 1 Department of Pharmacology, Sahlgrenska Academy at the University of Gothenburg, P.O. Box 431, 405 30 Gothenburg, Sweden; 2 Department of Neuroscience, Karolinska Institutet, Stockholm, Sweden; 3 Department of Clinical Neuroscience, Karolinska University Hospital, Stockholm, Sweden; 4 Aging Research Center, Karolinska Institutet, Stockholm, Sweden; 5 Department of Clinical Neuroscience and Rehabilitation, Sahlgrenska Academy at the University of Gothenburg, Gothenburg, Sweden; 6 Department of Clinical and Experimental Medicine, Faculty of Health Sciences, Linköping University, Linköping, Sweden; 7 Department of Neurology, Linköping University Hospital, Linköping University, Linköping, Sweden

**Keywords:** Parkinson’s disease, S100B, Single nucleotide polymorphism, Association study, Genotyping

## Abstract

**Background:**

In this study we investigated the association between SNPs in the S100B gene and Parkinson’s disease (PD) in two independent Swedish cohorts. The SNP rs9722 has previously been shown to be associated with higher S100B concentrations in serum and frontal cortex in humans. S100B is widely expressed in the central nervous system and has many functions such as regulating calcium homeostasis, inflammatory processes, cytoskeleton assembly/disassembly, protein phosphorylation and degradation, and cell proliferation and differentiation. Several of these functions have been suggested to be of importance for the pathophysiology of PD.

**Methods:**

The SNPs rs9722, rs2239574, rs881827, rs9984765, and rs1051169 of the S100B gene were genotyped using the KASPar® PCR SNP genotyping system in a case-control study of two populations (431 PD patients and 465 controls, 195 PD patients and 378 controls, respectively). The association between the genotype and allelic distributions and PD risk was evaluated using Chi-Square and Cox proportional hazards test, as well as logistic regression. Linear regression and Cox proportional hazards tests were applied to assess the effect of the rs9722 genotypes on age of disease onset.

**Results:**

The S100B SNPs tested were not associated with the risk of PD. However, in both cohorts, the T allele of rs9722 was significantly more common in early onset PD patients compared to late onset PD patients. The SNP rs9722 was significantly related to age of onset, and each T allele lowered disease onset with 4.9 years. In addition, allelic variants of rs881827, rs9984765, and rs1051169, were significantly more common in early-onset PD compared to late-onset PD in the pooled population.

**Conclusions:**

rs9722, a functional SNP in the 3’-UTR of the S100B gene, was strongly associated with age of onset of PD.

## Background

Sporadic Parkinson’s disease (PD) or idiopathic PD is the far most common form of PD and accounts for at least 90% of all cases. Among the suggested pathophysiological mechanisms of neurodegeneration in PD are increased generation of reactive oxygen species, mitochondrial pathology, and increase in intracellular calcium [1–3]. There is also evidence that immune and inflammatory mechanisms as well as impaired protein degradation are involved in the pathogenesis [4–6]. Recently, it was suggested that sporadic PD could be due to misfolded alpha-synuclein that spreads and by change reaction induce misfolding and pathological aggregation of native alpha-synuclein [7–9]. Conceivably, these mechanisms may operate simultaneously or in time sequence.

Regarding sporadic PD, a number of genetic polymorphism-based studies has been performed on a variety of candidate genes (see http://www.pdgene.org) [10]. In a recently performed meta-analysis of genome-wide association studies (GWAS) significance was obtained for 28 gene loci [11]. However, according to genome-wide complex trait analysis there are substantially more risk loci to be identified [12].

S100B is a highly conserved protein and a member of the S100 calcium-binding protein superfamily. It is expressed in various cell types in the central nervous system, such as astrocytes, neural progenitor cells, and various neuronal populations [13, 14], as well as in the enteric nervous system in glial cells important for the regulation of inflammation in the gut [15]. Being both intracellularly and extracellularly active, S100B has a wide range of functions. Within cells, the protein regulates calcium homeostasis, cytoskeleton assembly/disassembly, protein phosphorylation and degradation, and cell proliferation and differentiation [16]. Secreted S100B have paracrine, autocrine, and endocrine properties, modulating the activity of neurons, astrocytes, microglia, monocytes, and endothelial cells [16].

Elevated serum concentrations of S100B have been detected in several pathological conditions, such as acute brain injuries [17], schizophrenia [18, 19], and Alzheimer’s disease [20]. Regarding PD, conflicting results have been obtained. In one study S100B serum concentrations were not significantly different between PD patients and controls [21]. In another study, however, antibodies against S100B were detected in the blood of PD patients, but not in the control group [22]. Sathe et al. [23] recently showed significantly higher S100B concentrations post-mortem in substantia nigra of PD patients.

Animal studies suggest S100B to be involved in motor and memory functions. Transgenic mice overexpressing S100B showed symptoms similar to PD, exhibiting impaired motor coordination [24], whereas S100B knock-out mice have exhibited enhanced spatial ability and synaptic plasticity [25].

**Fig. 1:**
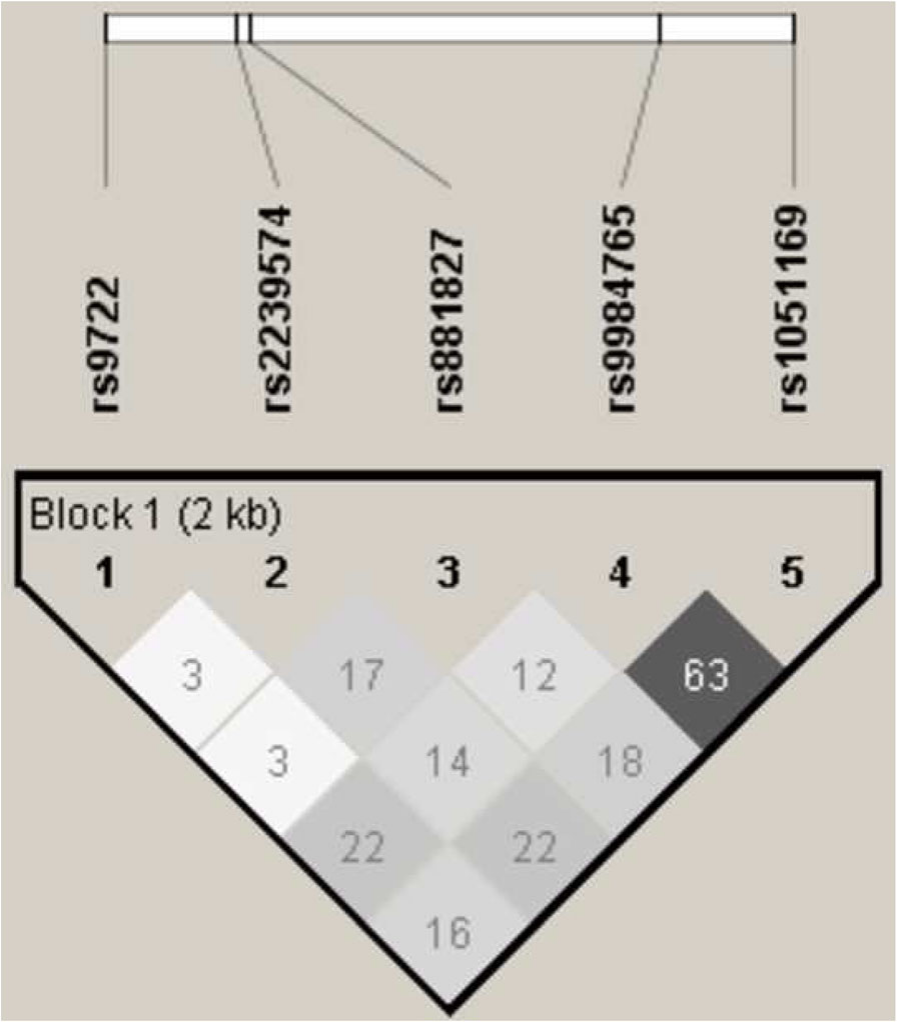
LD plot generated for the S100B gene using HaploView v.4.2

Considering these previous findings we performed a case-control study in two independent Swedish populations to evaluate the possible association between single nucleotide polymorphisms (SNPs) in the gene coding for S100B and PD. Since age of onset of PD seems to have a relatively high heritability, in one study estimated to be 40–60% [26], and previously have been reported to be associated with some gene polymorphisms [27], we also examined whether these SNPs affect age of onset of PD. We genotyped rs9722 and rs1051169 together with three other SNPs in the S100B gene, which were selected as Tag-SNPs (see Fig. 1).

## Methods

### Study populations

The discovery cohort consisted of 431 PD patients and 465 control subjects. The PD patients were recruited from four hospitals in Sweden (Gothenburg, Falköping, Skövde and Stockholm). Control subjects comprised unrelated outpatients in primary care in Gothenburg and participants in the Kungsholmen project, a community-based cohort in Stockholm of people aged 75 years and older [28]. Participants in the Kungsholmen project has been confirmed not to have PD. The validation cohort consisted of 195 PD patients and 378 control subjects. The PD patients were recruited from the hospitals in Linköping and Jönköping and control subjects were randomly collected from the population registry in the same recruitment area as for the hospitals. The patient and control groups in the validation cohort were frequency-matched by age and sex. All PD patients had been examined by neurologists and/or movement disorders specialists and fulfilled the Parkinson Disease Society Brain Bank criteria for idiopathic PD [29], except that the presence of more than one relative with the disease was not regarded as an exclusion criterion. Thirteen cases (3%) in the discovery cohort and 11 cases (5.6%) in the validation cohort had more than one affected relative. In the discovery cohort, 25% of the patients reported to have a first-, second- or third-degree relative with PD, and in the validation cohort, 21% of the patients report about having a first- or second degree relative with the disorder. Nearly all subjects (> 99%) were of Caucasian origin. All subjects had provided informed consent and the study were approved by the ethical committees at University of Gothenburg, Karolinska Institute and Linköping University.

Age of disease onset was defined as the time when the patients first noticed PD symptoms. The commonly used definition of having an “early age of onset” of PD if the disease begins at or before 50 years of age was used [6, 30, 31]. By using this definition, 87 patients (20%) in the discovery cohort and 25 patients (13%) in the validation cohort were categorized as having early-onset PD. The majority of the 87 early onset patients in the discovery cohort have previously been screened for mutations in the DJ-1, parkin, and PINK1 genes, and were not found be carriers of any of these [32, 33].

### Genotyping and statistical analysis

DNA from blood samples were genotyped using the KASPar® PCR SNP genotyping system (KBiosciences, Herts, UK). The three tag-SNPs were chosen by pair wise tagging (r^2^ ≥ 0.80) from the International HapMap Project database (release 27, Phase II + III, February 2009, on NCBI B36 assembly, dbSNP b126). Thirty-eight individuals in the discovery cohort and four individuals in the validation cohort were excluded due to poor DNA quality. Success rates for the investigated SNPs were between 96.6–99.6%. Differences in allelic distributions were analysed using a Chi-square test in Haploview 4.0 (Broad Institute, Cambridge, MA, USA) and logistic regression in SPSS 19.0 (IBM Corporation, Armonk, NY, USA) and multiple testing correction was carried out on the pooled data by Bonferroni procedures (analyses of 5 SNPs and 3 comparisons: controls versus all PD patients, controls versus early onset PD and early onset versus late onset PD; 15 tests, corrected significance level: *p* = 0.0033). Cox proportional hazard tests were performed in SPSS 19.0 on the whole population including controls, as well as on patients only. Age at disease onset was used for patients and age at examination was used for controls. The association between the SNPs and age at disease onset was also evaluated using linear regression in SPSS. For both Cox proportional hazards analysis and linear regression analysis, gender and sample group (discovery cohort or validation cohort) were used as covariates. The significance level was set at *p* = 0.05.

## Results

Demographic data of the populations are presented in Table 1. The genotype distributions of all five polymorphisms were in Hardy-Weinberg equilibrium in both control populations (*p*-value cut-off = 0.01). Three individuals were excluded in the statistical analysis of age of onset due to missing information about onset age. The allele and genotype distributions of the SNPs in Population 1 are displayed in Table 2.

**Table 1:** Demographic data describing the study populations

	Discovery cohort	Validation cohort	All
*N*	896	573	1469
Controls	465	378	843
Mean age	74.1	67.5	70.8
Males (%)	134 (28.8)	187 (49.5)	321 (38.1)
PD Patients	431	195	626
Mean age	67.6	71.4	69.5
Males (%)	258 (59.9)	121 (62.1)	379 (60.5)
Mean age of onset	59.3	63.6	61.5
Onset ≤50 y. of age (%)	87 (20.2)	25 (12.8)	112 (17.9)

**Table 2:** Allele and genotype frequencies of S100B SNPs in the discovery cohort

	Controls		Early onset PD		Late onset PD		*p*-Value^a^	*p*-Value^b^	OR^c^
	*n*	Frequency	*n*	Frequency	*n*	Frequency			
rs9722									
C	782	0.929	147	0.865	626	0.946	**2.0 × 10** ^**−4**^	**0.006**	**2.7 (1.6–4.7)**
T	60	0.071	23	0.135	36	0.054			
CC	365	0.867	64	0.753	296	0.894	**0.001**	**0.026**	
TC	52	0.124	19	0.224	34	0.103			
TT	4	0.010	2	0.024	1	0.003			
rs2239574									
C	559	0.661	118	0.686	463	0.704	0.654	0.522	1.1 (0.8–1.6)
T	287	0.339	54	0.314	195	0.296			
CC	188	0.444	38	0.442	168	0.511	0.202	0.320	
TC	183	0.433	42	0.488	127	0.386			
TT	52	0.123	6	0.070	34	0.103			
rs881827									
C	629	0.740	130	0.756	464	0.714	0.274	0.665	1.2 (0.8–1.8)
T	221	0.260	42	0.244	186	0.286			
CC	236	0.555	48	0.558	166	0.511	0.468	0.615	
TC	157	0.369	34	0.395	132	0.406			
TT	32	0.075	4	0.047	27	0.083			
rs9984765									
T	649	0.774	116	0.674	508	0.772	**0.008**	**0.005**	**1.6 (1.1–2.4)**
C	189	0.226	56	0.326	150	0.228			
TT	263	0.628	38	0.442	197	0.599	**0.028**	**0.004**	
CT	123	0.294	40	0.465	114	0.347			
CC	33	0.079	8	0.093	18	0.055			
rs1051169									
C	568	0.688	104	0.605	453	0.688	**0.037**	**0.035**	**1.4 (1.0–2.0)**
G	258	0.312	68	0.395	205	0.312			
CC	206	0.499	34	0.395	157	0.477	0.073	0.138	
GC	156	0.378	36	0.419	139	0.422			
GG	51	0.123	16	0.186	33	0.100			

Significant (*p* < 0.05) results are shown in bold. ^a^Early onset PD (≤50 years) compared to late onset PD (> 50 years). ^b^Early onset PD compared to controls. ^c^OR (95% confidence interval) for early onset PD compared to controls. *P*-values were calculated from Chis-square test and ORs were calculated using logistic regression

In the discovery cohort, no significant differences in allelic or genotype frequencies were observed for any of the SNPs when comparing PD patients and controls, using Chi-square test and logistic regression analysis (results not shown). However, there were significant differences in allelic and genotype frequencies for several SNPs in the S100B gene when comparing PD patients with an early age of onset (≤50 years) to PD patients with a late disease onset as well as when comparing to controls. The T allele of rs9722, the C allele of rs9984765, and the G allele of rs1051169 were significantly more common in early-onset PD patients compared to late-onset PD patients (*p* = 0.0002, *p* = 0.008, and *p* = 0.037, respectively) and when compared to controls (*p* = 0.006, *p* = 0.005, and *p* = 0.035, respectively). Furthermore, the genotype frequencies of rs9722 and rs9984765 differed significantly when comparing early-onset PD patients and late-onset PD patients (*p* = 0.001 and *p* = 0.028, respectively) as well as when comparing early-onset PD patients and controls (*p* = 0.026 and *p* = 0.004, respectively).

To replicate these findings, we genotyped the S100B SNPs in an independent validation cohort (see Table 3). No significant differences in allele or genotype frequencies were observed for any of the SNPs when comparing all PD patients with controls in the validation cohort. However, the T allele of rs9722, and the C allele of rs881827 were significantly more common in early-onset than late-onset PD (*p* = 0.005 and *p* = 0.014, respectively) and the C allele of rs881827 was significantly more common in early-onset PD than in controls (*p* = 0.035). The genotype frequencies of rs9722 differed significantly when comparing early-onset to late onset PD patients (*p* = 0.021).

**Table 3:** Allele and genotype frequencies of S100B SNPs in the validation cohort

	Controls		Early onset PD		Late onset PD		*p*-Value^a^	*p*-Value^b^	OR
	*n*	Frequency	*n*	Frequency	*n*	Frequency			
rs9722									
C	679	0.913	37	0.841	325	0.950	**0.005**	0.109	**3.6 (1.4–9.3)**
T	65	0.087	7	0.159	17	0.050			
CC	312	0.839	16	0.727	157	0.918	**0.021**	0.277	
TC	55	0.148	5	0.227	11	0.064			
TT	5	0.013	1	0.045	3	0.018			
rs2239574									
C	515	0.689	27	0.643	238	0.704	0.415	0.535	1.3 (0.7–2.6)
T	233	0.311	15	0.357	100	0.296			
CC	172	0.460	9	0.429	86	0.509	0.731	0.635	
TC	171	0.457	9	0.429	66	0.391			
TT	31	0.083	3	0.143	17	0.101			
rs881827									
C	537	0.718	38	0.864	240	0.702	**0.024**	**0.035**	**2.7 (1.1–6.6)**
T	211	0.282	6	0.136	102	0.298			
CC	200	0.535	17	0.773	85	0.497	0.051	0.093	
TC	137	0.366	4	0.182	70	0.409			
TT	37	0.099	1	0.045	16	0.094			
rs9984765									
T	549	0.736	31	0.705	260	0.760	0.420	0.647	1.3 (0.7–2.7)
C	197	0.264	13	0.295	82	0.240			
TT	202	0.539	11	0.500	104	0.608	0.587	0.883	
CT	148	0.395	9	0.409	52	0.304			
CC	25	0.067	2	0.091	15	0.088			
rs1051169									
C	475	0.656	26	0.591	224	0.663	0.346	0.378	1.4 (0.7–2.6)
G	249	0.344	18	0.409	114	0.337			
CC	156	0.430	9	0.409	77	0.456	0.469	0.284	
GC	165	0.455	8	0.364	70	0.414			
GG	42	0.116	5	0.227	22	0.130			

Significant (*p* < 0.05) results are shown in bold. ^a^Early onset PD (≤50 years) compared to late onset PD (> 50 years). ^b^Early onset PD compared to controls. ^c^OR (95% confidence interval) for early onset PD compared to controls. *P*-values were calculated from Chis-square test and ORs were calculated using logistic regression

**Fig. 2:**
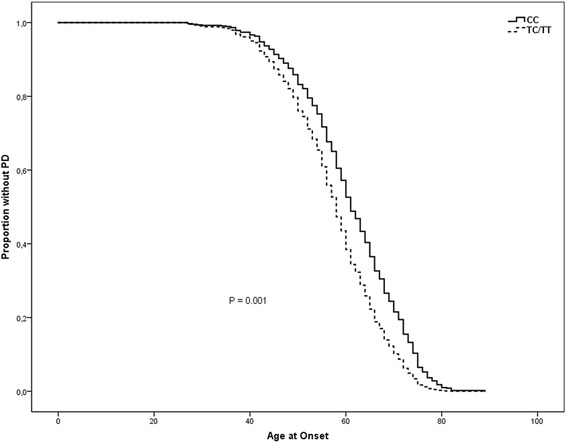
Age of onset of PD stratified by genotypes of the SNP rs9722, adjusted for gender and sample group

The allelic frequencies for the pooled populations are presented in Table 4, showing significant differences for all SNPs, except rs2239574. Notable is the highly significant allelic and genotype frequency difference of the rs9722 SNP in early-onset and late-onset PD (*p* = 0.0000041 and *p* = 0.00005, respectively (Bonferroni corrected *p*-values: *p* = 0.0000615 and *p* = 0.00075, respectively)). In line with these results, a Cox regression analysis comprising all PD patients in the pooled population (see Fig. 2) confirmed that the T-allele of rs9722 is associated with an earlier age of onset (HR = 1.49; 95% C.*I* = 1.17–1.90, *p* = 0.001). The Cox proportional hazards tests were insignificant when analyzing the whole population including controls (results not shown). Furthermore, when analyzing the data for the pooled population, linear regression showed that disease onset was significantly lower with more T alleles of the rs9722 polymorphism (*p* = 0.00004) and that each T allele lowered disease onset with 4.9 years. In addition, the haplotype TCCCG of the five genotyped SNPs (rs9722, rs2239574, rs881827, rs9984765, rs1051169) was also significantly more common in early- onset compared to late-onset PD in both populations (*p* = 0.01 for the discovery cohort, *p* = 0.035 for the validation cohort, and *p* = 0.000019 for both combined) as well as in early onset PD as compared to controls (*p* = 0.0099 for the discovery cohort, *p* = 0.064 for the validation cohort, and *p* = 0.0031 for both combined).

**Table 4:** *P*-values of comparisons of S100B SNPs on allele and genotype level in the pooled population

	rs9722	rs2239574	rs881827	rs9984765	rs1051169
*p*-Value^a^ allele	*4.1 × 10 ^**−6**^	0.448	**0.043**	**0.007**	**0.028**
*p*-Value^a^ genotype	*5.0 × 10 ^**−5**^	0.233	0.131	**0.018**	**0.049**
*p*-Value^b^ allele	*0.003	0.912	0.132	**0.016**	**0.038**
*p*-Value^b^ genotype	**0.015**	0.731	0.289	**0.034**	0.079
OR^c^	**2.9 (1.8–4.7)**	1.1 (0.8–1.6)	**1.4 (1.0–2.0)**	**1.6 (1.1–2.1)**	**1.4 (1.0–1.9)**

^a^Early onset PD (≤50 years) compared to late onset PD (> 50 years). ^b^Early onset PD compared to controls. ^c^OR (95% confidence interval) for early onset PD compared to controls. Significant (*p* < 0.05) results are shown in bold. With Bonferroni correction for multiple testing (analyses of 5 SNPs and 3 comparisons; controls versus all PD patients, controls versus early onset PD and early onset versus late onset PD) significant (*p* < 0.0033) results are shown by *. *P*-values were calculated from Chis-square test and ORs were calculated using logistic regression

## Discussion

In the present study rs9722, rs9984765 and rs10511669 in the S100B gene were associated with age of onset of PD in Population 1 and rs9722 and rs881827 were associated with age of onset in Population 2. In both populations, the T allele of rs9722 was strongly associated with early-onset PD. Furthermore, in both populations, the haplotype TCCCG of the five genotyped SNPs was more frequent in early-onset than in late-onset PD.

Several of the large GWAS studying PD [10] include SNPs in the S100B gene, although only one study genotyped the SNPs investigated in the present study [34]. However, other GWAS include SNPs in high LD with the ones genotyped in our study, which makes it possible to impute the genotypes of our SNPs. None of these studies report significant associations regarding PD and SNPs in the S100B gene, which is in line with the findings in the present study.

Noteworthy, only two GWAS so far have investigated age at onset of PD [35, 36]. In both studies, not rs9722 itself, but SNPs in high LD with rs9722, were genotyped and no significant association with age of onset were found. However, to be able to compare the results from different association studies it is important that the inclusion criteria for patients and controls used in the studies are similar, especially when searching for genetic risk variants of low impact in a complex disorder like PD. In the GWAS studying onset age in PD by Latourelle et al. [35]*,* two of three of the PD populations investigated include samples recruited to study familial PD which means that all of the patients in those populations have a family history of the disease, making their sample different from ours where at least 80% of the patients are sporadic cases. Furthermore, in the GWAS by Spencer et al. [36]*,* there is a quite large difference in mean age of onset (65.8 years) compared to our study (61.5 years). These dissimilarities might be part of the explanation to the deviation in results when comparing these two studies with the present one. Furthermore, the diversity of ethnicity might also be of importance and the populations studied in the present paper are very homogenous in that regard.

The S100B gene was investigated in a study of PD patients by Guo et al. [37]. The authors screened a Chinese PD-population for mutations in the coding parts of the gene, and consequently only one of the SNPs investigated in the present study, rs1051169, was possible to detect. The frequency found for this SNP was quite similar to the frequencies of it in our Caucasian PD patients.

It has been proposed that S100B has neurotrophic or neurotoxic properties depending on the extracellular concentration [16]. In normal conditions, S100B in nanomolar concentrations seems to protect neurons against oxidative stress [38, 39]. However, at higher extracellular concentrations, it may act as a pro-inflammatory substance activating astrocytes and microglia and inducing apoptosis [40–42]. Alternatively, S100B at high concentrations merely is a secondary reactive phenomena or marker of inflammation intensity rather than promoting inflammation (for discussion see Lam et al. [43]).

Parts of the effects of S100B appear to be mediated by the receptor for advanced glycation end products (RAGE) [44, 45]. In neurons, nanomolar concentrations of S100B promote cell survival by RAGE-mediated NF-KB activation, leading to upregulation of the anti-apoptotic factor Bcl-2 [39, 46, 47]. However, in micromolar concentrations, the RAGE-mediated S100B toxic effects are due to overproduction of reactive oxygen species (ROS) [44], leading to apoptosis.

The findings that high concentrations of S100B could have neurotoxic effects are especially interesting, because the rs9722 SNP, located in the 3′ untranslated region (3´-UTR), appears to be functional in that healthy individuals with the T allele variant, the variant we found to be more common in PD with early onset, have been reported to have higher serum and frontal cortex concentrations of S100B [48]. Furthermore, functional studies of peripheral blood mononuclear cells from healthy volunteers show that cells with the CT genotype of rs9722 express more than twice the amount of S100B mRNA as well as S100B protein as compared to cells with the CC genotype [49].

The allelic and genotype frequencies of the S100B polymorphisms were similar in late-onset PD and controls, but early-onset patients differed to both late-onset patients and controls. Interestingly, a newly published study analyzing data from a meta-analysis of GWAS found support for that an individual’s polygenic risk score were higher in PD patients with early onset as compared to those with late onset [50]. This pattern suggests that early-onset patients may have a different pathophysiology compared late-onset patients, with the S100B allelic variants conferring a risk only for early-onset patients. Support for this view comes from the observation that the incidence and prevalence of PD after the age of 50 increases almost exponentially in contrast to early onset PD [51], which besides is the basis for using this age as a cut-off while defining early age of onset PD.

However, another interpretation is that the functional activity of S100B does not influence the risk to be affected of neither PD with a late onset nor PD with an early onset but rather modulates the age of onset of PD, a notion that is further supported by the linear regression analysis and Cox proportional hazards tests performed in the present paper regarding the rs9722 SNP. Moreover, the observation that the genetic influence on the risk for sporadic PD, as judged by the very low concordance rate in monozygotic twins [52, 53], suggests that environmental factors are most important for causing the disease.

The distinction between gene variants influencing the risk to be affected by PD and variants that modify age of onset might be biologically significant; risk variants point to the initial cause, whereas onset modifiers implicate the processes that begin after the initial insult affecting the threshold for developing clinical signs [54]. Interestingly, segregation analyses of PD suggest stronger evidence for major genes influencing age of onset than for genes influencing susceptibility to disease [55, 56].

## Conclusions

Even though the population sizes used in the current study are quite small, the results suggest that S100B activity could influence age of onset of sporadic PD. By resulting in higher S100B levels, the minor allele of the SNP rs9722 might modulate age of onset, potentially by activation of inflammatory processes or by increasing intracellular calcium.

## Abbreviations

GWAS: Genome-wide Association Study
PD: Parkinson’s disease
RAGE: Glycation End Products
SNP: Single Nucleotide Polymorphism
